# Synergy and Antagonism of Active Constituents of ADAPT-232 on Transcriptional Level of Metabolic Regulation of Isolated Neuroglial Cells

**DOI:** 10.3389/fnins.2013.00016

**Published:** 2013-02-20

**Authors:** Alexander Panossian, Rebecca Hamm, Onat Kadioglu, Georg Wikman, Thomas Efferth

**Affiliations:** ^1^Swedish Herbal Institute Research and DevelopmentGöteborg, Sweden; ^2^Department of Pharmaceutical Biology, Institute of Pharmacy and Biochemistry, Johannes Gutenberg UniversityMainz, Germany

**Keywords:** pharmacogenomics, *Rhodiola rosea*, *Schisandra chinensis*, *Eleutherococcus senticosus*, ADAPT-232, salidroside, eleutheroside E, schizandrin B

## Abstract

Gene expression profiling was performed on the human neuroglial cell line T98G after treatment with adaptogen ADAPT-232 and its constituents – extracts of *Eleutherococcus senticosus* root, *Schisandra chinensis* berry, and *Rhodiola rosea* root as well as several constituents individually, namely, eleutheroside E, schizandrin B, salidroside, triandrin, and tyrosol. A common feature for all tested adaptogens was their effect on G-protein-coupled receptor signaling pathways, i.e., cAMP, phospholipase C (PLC), and phosphatidylinositol signal transduction pathways. Adaptogens may reduce the cAMP level in brain cells by down-regulation of adenylate cyclase gene ADC2Y and up-regulation of phosphodiesterase gene PDE4D that is essential for energy homeostasis as well as for switching from catabolic to anabolic states and vice versa. Down-regulation of cAMP by adaptogens may decrease cAMP-dependent protein kinase A activity in various cells resulting in inhibition stress-induced catabolic transformations and saving of ATP for many ATP-dependant metabolic transformations. All tested adaptogens up-regulated the PLCB1 gene, which encodes phosphoinositide-specific PLC and phosphatidylinositol 3-kinases (PI3Ks), key players for the regulation of NF-κB-mediated defense responses. Other common targets of adaptogens included genes encoding ERα estrogen receptor (2.9–22.6 fold down-regulation), cholesterol ester transfer protein (5.1–10.6 fold down-regulation), heat shock protein Hsp70 (3.0–45.0 fold up-regulation), serpin peptidase inhibitor (neuroserpin), and 5-HT3 receptor of serotonin (2.2–6.6 fold down-regulation). These findings can be reconciled with the observed beneficial effects of adaptogens in behavioral, mental, and aging-associated disorders. Combining two or more active substances in one mixture significantly changes deregulated genes profiles: synergetic interactions result in activation of genes that none of the individual substances affected, while antagonistic interactions result in suppression some genes activated by individual substances. These interactions can have an influence on transcriptional control of metabolic regulation both on the cellular level and the level of the whole organism. Merging of deregulated genes array profiles and intracellular networks is specific to the new substance with unique pharmacological characteristics. Presumably, this phenomenon could be used to eliminate undesirable effects (e.g., toxic effects) and increase the selectivity of pharmacological intervention.

## Introduction

The term adaptogen was introduced in scientific literature in the 1950s to refer to substances that increase the “state of non-specific resistance” under stress conditions (Lazarev, 1958; Lazarev et al., 1959). The adaptogen concept was based on the theory of stress (Selye, 1950), initially defined as a state of threatened homeostasis, and a general adaptation syndrome characterized by a non-specific response of the organism to diverse stressors (physical, emotional, environmental, etc.). It has been postulated that adaptogens should be safe and are able to normalize body functions irrespective of the nature of stressors (Brekhman II and Dardymov, 1969). On the verge of the new millennium, the definition of adaptogens was updated to “*a new class of metabolic regulators which increase the ability of an organism to adapt to environmental factors and to avoid damage from such factors*” (Panossian et al., 1999a). The concept of adaptogens is now generally accepted by the scientific community (EMEA/HMPC/102655/, 2007; Samuelsson and Bohlin, 2009), although it has yet to gain prominence in mainstream pharmacology. In this context recent research progress has taken two main forms:

- Convincing clinical evidence has been generated on the efficacy of adaptogens, based on clinical trials performed in accordance with the International Conference in Harmonization Regulations (ICH) standards for good clinical practice (Davydov and Krikorian, 2000; Goulet and Dionne, 2005; Sarris, 2007; Bleakney, 2008; Panossian and Wikman, 2009, 2010; Dwyer et al., 2011; Hung et al., 2011; Iovieno et al., 2011; Sarris et al., 2011; Chan, 2012; Ishaque et al., 2012),
- Some key molecular mechanisms of adaptogen activity were defined (Panossian et al., 2007, 2009, 2012).

The stress-protective activity of adaptogens is associated with regulation of homeostasis both on:

- the system level *via* several mechanisms of action which are linked to the hypothalamic-pituitary-adrenal (HPA) axis, and the cellular level *via* activation of molecular chaperones, mainly hsp70 proteins, and the regulation of key mediators of the stress response, including neuropeptide Y (NPY), cortisol, nitric oxide, stress-activated protein kinase JNK, and forkhead box O transcription factor (Panossian et al., 2007, 2009, 2012; Wiegant et al., 2009).

An important role of CNS system in stress is generally accepted, since the stress concept has been defined by Selye (Fink, 2000). Effect of adaptogens on CNS system, particularly neuroprotective activity has been demonstrated in many animal and human studies (Panossian and Wikman, 2010; Panossian et al., 2011). Clinical efficacy of adaptogens in behavioral and mental disorders such as depression, anxiety, bipolar disorder, chronic, and stress-induced fatigue has been recently reviewed (Panossian and Wikman, 2009, 2010).

ADAPT-232 (Chisan^®^) is a traditional herbal medicinal product consisting of a fixed combination of extracts from *Rhodiola rosea* root, *Schisandra chinensis* berry, and *Eleutherococcus senticosus* root. It is taken for decreased body performance such as fatigue and weakness (Bogatova et al., 1997; Narimanian et al., 2005; Aslanyan et al., 2010). In general, herbal mixtures exert their bioactivities through synergistic interactions of single components. This synergism can be attributed to the fact that medicinal herbs contain many different phytochemicals, which may mutually influence each other’s activity. More than 140 compounds have been identified in *R. rosea* roots (Panossian and Wikman, 2010), 100 compounds in *E. senticosus* roots (Huang et al., 2011), and about 200 compounds in *S. chinensis* berries (Panossian and Wikman, 2008). Many of them were shown to be active in pharmacological *in vivo* and *in vitro* experiments, and likely contribute to the activity of the total extracts (Wagner et al., 1994; Panossian, 2003; Panossian and Wagner, 2005, 2011; Panossian and Wikman, 2008, 2010; Panossian et al., 2008; Huang et al., 2011). It has been shown that the adaptogenic activity of ADAPT-232 is associated with key mediators of stress response, e.g., heat-shock-proteins (Hsp70) and NPY, involved in the regulation of homeostasis, oxidative stress, energy metabolism, cognitive function, and activation of the immune system during fatigue and exhaustion (Prodius et al., 1997; Panossian et al., 2009). However, the cellular and molecular modes of action are not well understood due to the fact that herbal remedies in general have multiple targets and several mechanisms rather than a single mechanism may account for their pharmacological effects.

Any pharmacological effect represents an integrated response of an organism to the drug. The response can be associated with interactions between the drug and the cell at various levels of regulation:

- The level of a small physiologically active molecules, e.g., cAMP plays an important role in the integrative response of the organism. This is the so-called metabolomic level, because ATP is a precursor of cAMP and AMP is a metabolite.
- The level of proteins involved in the synthesis or degradation of ligands or ligand receptors. This is the so-called proteomics level of regulation.
- Genes encoding proteins involved in synthesis, degradation, signal reception, and regulation. This is the so-called genomic and transcriptional level of regulation.

Activation or suppression of gene expression results in activation or inhibition of the biosynthesis of encoded proteins (enzymes, cofactors, or receptors), which in turn affects the production and function of active small molecules. Gene expression profiles aid in tracing molecular interactions and signal transduction pathways and to predict the pharmacological effect a drug will have on various cellular functions.

Recently, the so-called “-omics” technologies have been established for the analysis of pharmacological effects (Ulrich-Merzenich et al., 2007; Sarris et al., 2012). Since transcriptomics and proteomics can monitor cellular changes in gene or protein expression upon drug treatment in a comprehensive fashion, these techniques may be exquisitely suited to investigate the multi-faceted mechanisms of herbal formulas. Therefore, we have analyzed the microarray-based transcriptome-wide mRNA expression profiles of the neuroglial cell line T98G after exposure to ADAPT-232 and its herbal components, *R. rosea*, *S. chinensis*, and *E. senticosus*.

The choice of neuroblastoma cell line T98G for our study is based on results obtained in many publications (Stein, 1979; Guzhova et al., 2001; Su et al., 2012). In the central nervous system, approximately 90% of the cells are glia. Glia has been shown to have several functions, including serving as a transportation link between the bloodstream and neurons, uptake of neurotransmitters, synthesis and release of neurotrophic factors, immune regulation, and modulation of synaptic activity (Henn and Hamberger, 1971; Haydon, 2001; Ullian et al., 2001). Glia contributes to the defense of the brain through the expression of the innate immune response, promoting the clearance of neurotoxic proteins and apoptotic cells from the CNS as well as by regulating the entry of inflammatory systemic cells into the brain at the blood-brain barrier (Nguyen et al., 2002; Hauwel et al., 2005). This stimulates both tissue repair and the rapid restoration of tissue homeostasis. An important physiological function of neuroglial cells is metabolic supply of energy and other substances, maintaining brain homeostasis – a function supposed to be the characteristic for adaptogens by definition. Glial cell express a variety of hormonal receptors, which are critical during stress-induced diseases. Glial cell express steroid receptors and generate many steroid hormones which elicit rapid non-genomic effects on neurons via both membrane-bound G-protein coupled receptors and nuclear genomic receptors known to activate RNA and protein synthesis (Bennett, 2000). Guzhova et al. (2001) show that glioma cells export Hsp70 into the culture medium whether under normal conditions or subjected to heat shock. Hsp70 can be released by glia and that exogenous Hsp70 can enhance neuronal stress tolerance (Guzhova et al., 2001). Finally, an ability of astrocytes, but not neurons, to prevent macrophage and T-Cell inflammation in the CNS, to attenuate axonal loss and gliosis resulting in neuroprotection in experimental autoimmune encephalomyelitis, the most widely used mouse model of multiple sclerosis (Spencea et al., 2011) was the reason to select neuroglia cells in our studies devoted to understand molecular mechanisms of action of adaptogens.

**Figure 1:**
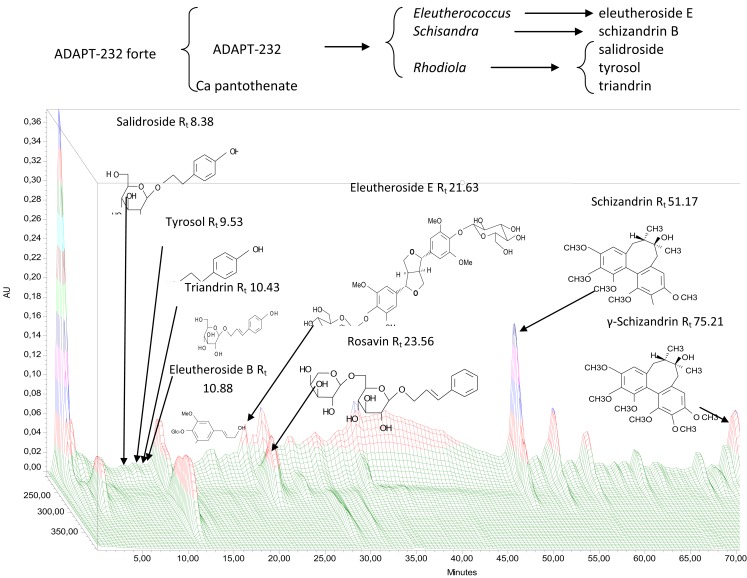
**Experimental setting for microarray-based transcriptome-wide mRNA profiling and 3D-HPLC fingerprint of ADAPT-232**.

The aim of the investigation was to compare similarities and differences in gene expression profiles upon treatment with these three adaptogenic plants. We compared the two plant extracts ADAPT-232 and ADAPT-232 forte (which is the same composition as ADAPT-232 except also includes the anti-stress vitamin, calcium pantothenate). We also compared these two fixed-composition mixtures to individual active compounds isolated from these plants, namely, salidroside, triandrin, tyrosol, eleutheroside E, and schizandrin B (Figure 1). This pharmacogenomic analysis facilitates increased understanding of the molecular modes of action of adaptogens and the synergism of ADAPT-232 forte.

## Materials and Methods

### Drugs and chemicals

Eleutheroside E, schizandrin B, salidroside, and tyrosol, were purchased from Chromadex (Irvine, CA, USA). Triandrin was isolated in Swedish Herbal Institute (SHI) Research and Development (Göteborg, Sweden), and identified by comparison (HPLC, TLC, UV) with the authentic reference sample kindly provided by Prof. Zapesoznaya (Institute of Officinal and Aromatic plants VILAR, Moscow, Russia). Pharmaceutical grade standardized extracts of *R. rosea* L. roots, *E. senticosus* (Rupr. et Maxim) Harms roots, and *S. chinensis* (Turcz.) Baill. berries, were manufactured in accordance to ICH Q7A and EMEA guidelines for Good Agricultural and Collecting Practice (GACP) and Good Manufacturing Practice (GMP) of active pharmaceutical ingredients (API). We have previously described the detailed composition and chemical characterization of ADAPT-232 and ADAPT-232 forte (Panossian et al., 2009, 2012; Aslanyan et al., 2010). The content of plant extracts and their active markers was the same in both adaptogen preparations; the only difference was the presence of calcium pantothenate in ADAPT-232 forte. Working samples used in experiments were prepared by dilution of stock solutions (5 mg/mL) of ADAPT-232, ADAPT-232 forte, Radix Eleutherococci genuine extract, Radix Rhodiola genuine extract, Fructus Schisandrae genuine extract or 10 mM solutions of purified analytical markers – salidroside (3 mg/mL), triandrin (3.1 mg/mL), eleutheroside E (7.4 mg/mL), schizandrin B (4 mg/mL), or tyrosol (1.4 mg/mL) with appropriate volumes of phosphate buffered saline solution (PBS). Working solutions of 200 μL were added to 3 mL of cell culture to obtain the same final concentrations of active markers and genuine extract as in the incubation media of ADAPT-232 and ADAPT-232 forte (Table 1). An exception was test sample B, where the concentration was 100-fold lower than in test sample A.

**Table 1:** **Concentrations used to treat T98G neuroglial cells for microarray experiments**.

Drug	Concentration	Designation
ADAPT-232 forte	300 μg/mL	Test sample A
ADAPT-232 forte	3 μg/mL	Test sample B
ADAPT-232	172 μg/mL	Test sample C
*Eleutherococcus* extract	30 μg/mL	Test sample D
*Schisandra* extract	100 μg/mL	Test sample E
*Rhodiola* extract	40 μg/mL	Test sample F
Eleutheroside E	0.67 μM	Test sample G
Schisandrin B	5 μM	Test sample H
Salidroside	3 μM	Test sample I
Triandrin	1.5 μM	Test sample J
Tyrosol	3 μM	Test sample K

The dose of 300 μg/mL (final concentration of ADAPT-232 forte in incubation media) is based on the results of a recent pharmacokinetic study of *R. rosea*-derived salidroside in human blood plasma, where we measured concentrations of ∼1 μg/mL = 3 μM (Panossian et al., 2010). However, in recent *in vitro* experiments, an up-regulation of HSF1, Hsp72, and NPY was observed at 100-fold lower concentrations of ADAPT-232 forte (Panossian et al., 2012). Therefore, in the present investigation ADAPT-232 forte was tested in two concentrations, 300 and 3 μg/mL (Table 1).

The concentrations of the total extracts of three herbal ingredients and their active constituents are compatible is all test samples: e.g., final concentration of salidroside is the same – 3 μM (900 μg/L) in all test samples containing salidroside, namely in Rhodiola extract, ADAPT-232, and ADAPT-232 forte. Similarly – schizandrin B, eleutheroside E triandrin, and tyrosol, their concentrations were calculated based of the results of HPLC analysis of their content in genuine extracts and their combinations. The concentrations of genuine extracts have been calculated using specifications of their combinations (ADAPT-232 and ADAPT-232 forte) to ensure that they corresponds to therapeutically effective doses.

### Cell culture

The human neuroglial cell line T98G was purchased from the American Type Culture Collection (ATCC, CRL-1690). Cells were grown in DMEM + GlutaMAX-I (Gibco, Darmstadt, Germany) with 10% fetal bovine serum (Gibco, Darmstadt, Germany) and 1% penicillin/streptomycin (Gibco, Darmstadt, Germany). They were passaged twice a week and maintained in a 37°C incubator in a humidified atmosphere with 5% CO_2_. All experiments were conducted using cells in the logarithmic growth phase.

### Drug treatment

T98G cells were seeded 24 h before treatment on 6-well plates in a density of 150,000 cells per well. The next day, old medium was removed and cells were treated in a final volume of 3 mL (Table 1).

The ethanol content was 0.8% for cells treated with isolated compounds and vehicle-treated control cells (test sample Z). Two technical replicates were performed for each sample. Cells were incubated with the test substances for 24 h at 37°C and then subjected to RNA isolation.

### mRNA isolation and quality control

Cells were harvested after 24 h of treatment. Total RNA was isolated using InviTrap Spin Universal RNA Mini kit (Stratec Molecular, Berlin, Germany) and dissolved in RNAse free-water. The RNA of the two technical replicates was pooled (1:1) resulting in one sample for each treatment/control. The quality of total RNA was checked by gel analysis using the Total RNA Nano chip assay on an Agilent 2100 Bioanalyzer (Agilent Technologies GmbH, Berlin, Germany). All samples were of highest quality with RIN values of 10.

### Gene expression profiling

Microarray hybridizations were performed at the Institute of Molecular Biology (Mainz, Germany). Whole Human Genome RNA chips (*860K Agilent*) were used for gene expression profiling. Probe labeling and hybridization procedures were carried out following the One-Color Microarray-Based Gene Expression Analysis Protocol^1^. Briefly, total RNA was labeled and converted to cDNA. Then, fluorescent cRNA (Cyanine 3-CTP) was synthesized and purified using the QIAgen RNeasy Kit. After fragmentation of the cRNA, samples were hybridized for 17 h at 65°C. Microarray slides were washed and scanned with the Agilent Microarray Scanning system. Images were analyzed and data was extracted. The background was subtracted and data was normalized using the standard procedures of Agilent Feature Extraction Software.

### Microarray data analysis

Expression data was further analyzed using Chipster software^2^ to filter genes by varying expression and significance. These steps include filtering genes to isolate those that were up- or down-regulated by one to three times the standard deviation (depending on the total number of extremely up- or down-regulated genes). A subsequent assessment of significance using empirical Bayes *t*-test further narrowed the pool of genes. All genes further considered showed a significant difference from the control with *p*-value < 0.05, or otherwise are noted. Finally, filtered data was used in Ingenuity pathway analysis for Core analysis, in order to determine networks and pathways influenced by the drug treatments^3^.

### Real-Time RT-PCR

Validation of the microarray data was done by real-time RT-PCR for five selected genes which were tested for two drug-treated samples (Tables 2 and 3). PCR primers were designed using Roche Universal Probe Design^4^ and GenScript Real-Time PCR Primer Design^5^ tools. Amplification specificities were checked with Primer Blast^6^ using the sequence data from the NCBI RefSeq Human mRNA data base. Primer nucleotide sequences and used primer concentrations are shown in Table 2. Oligonucleotides were synthesized by MWG Eurofins. Extracted RNA was converted to cDNA with RevertAid H Minus First Strand cDNA Synthesis Kit (Thermo Scientific). Real-time RT-PCR experiments were performed with the Roche Bio-Rad CFX384 real-time PCR detection system. Total reaction volume was 20 μL. About 4 μL 5x Hot Start Taq EvaGreen qPCR Mix (no ROX; Axon), 75–250 nM final primer concentration, and about 500 ng RNA (converted to cDNA) were used per reaction. The protocol was as follows: 50, 0°C for 2 min, 95, 0°C for 10 min, 45 cycle of 95, 0°C for 15 s, Annealing Temperature for 1 min, 72, 0°C for 1 min and 95, 0°C for 1 min. The housekeeping gene RPS13 served as reference for standardization. All measurements were done in duplicates to calculate mean values and standard deviations. Standardized *C_t_* (cycle threshold) values for the genes in samples were obtained by dividing the *C_t_* values of genes in drug-treated samples by *C_t_* values of RPS13 gene and multiplying with the *C_t_* value of RPS13 in the control. Fold changes were calculated with the Δ*C_t_* (Standardized *C_t_* of the gene in drug-treated sample – *C_t_* of the gene in untreated sample) method where the fold change is equal to $2^{-\Delta C_{t}}$ for the up-regulated genes and $-(2^{-\Delta C_{t}})$ for the down-regulated genes.

**Table 2:** **Primer nucleotide sequences and used primer concentrations**.

Target gene	Sequence	Concentration (nM)	Annealing temperature (°C)
ADCY2	Fw: 5′-CTGCTCGCCGTCTTCTTCGCG-3′	125	57.9
	Rev: 5′-CGCCAGGGCAGTTGGAACTGTTAT-3′		
CETP	Fw: 5′-GAGACTGCCAAGGTGATCCAGA-3′	125	58
	Rev: 5′-GTGGTGTAGCCATACTTCAGGG-3′		
ESR1	Fw: 5′-CACCCAGGGAAGCTACTGTTTG-3′	125	58
	Rev: 5′-ATCTCCACCATGCCCTCTACAC-3′		
HTR3D	Fw: 5′-TGACTGTTCTGCTGGGCTACA-3′	125	58
	Rev: 5′-GCGAAGTAGACACCTCGCTT-3′		
PDE4D	Fw: 5′-GAATCAGAGAACATTCAACGACCAACCAG-3′	75	63.4
	Rev: 5′-GCAGATGTGCCATTGTCCACATCAAAA-3′		
RPS13	Fw: 5′-GGTTGAAGTTGACATCTGACGA-3′	250	57.9–63.4
	Rev: 5′-CTTGTGCAACACATGTGAAT-3′		

**Table 3:** **Validation of microarray-based mRNA expression by quantitative real-time RT-PCR**.

Gene	Sample	Method	Untreated cells	Treated cells	FC (log)	FC
ADCY2	ADAPT-232 forte*	Microarray	1,00	1,00	0	1,00
		Real-time RT-PCR				1,08
	*Schisandra* extract	Microarray	1,00	1,00	0	1,00
		Real-time RT-PCR				1,04
CETP	ADAPT-232 forte*	Microarray	1,00	1,00	0	1,00
		Real-time RT-PCR				1,52
	*Eleutherococcus* extract	Microarray	8,75	6,13	−2,62	−6,15
		Real-time RT-PCR				−1,21
ESR1	ADAPT-232 forte**	Microarray	8,23	4,63	−3,60	−12,13
		Real-time RT-PCR				−1,74
	ADAPT-232 forte*	Microarray	1,00	1,00	0	1,00
		Real-time RT-PCR				1,42
HTR3D	*Eleutherococcus* extract	Microarray	6,93	5,65	−1,28	−2,43
		Real-time RT-PCR				−2,75
	Salidroside	Microarray	6,93	4,21	−2,72	−6,59
		Real-time RT-PCR				−2,81
PDE4D	ADAPT-232	Microarray	1,00	1,00	0	1,00
		Real-time RT-PCR				1,44
	*Rhodiola* extract	Microarray	4,84	6,85	2,01	4,03
		Real-time RT-PCR				2,91

*Concentration 3 μg/mL, **Concentration 300 μg/mL.

The correlation coefficient between mRNA expression values determined by microarray hybridization and real-time RT-PCR was *R* = 0.81 (Pearson Correlation Test), Table 3.

## Results

**Figure 2:**
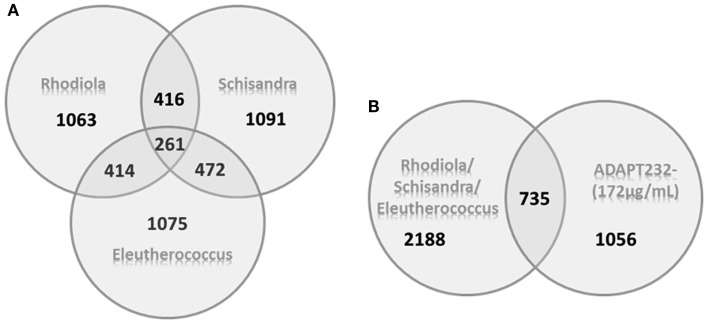
**Venn diagrams of deregulated genes induced by the treatment of neuroglial cells with *Rhodiola rosea* root, *Schisandra chinensis* berries, and *Eleutherococcus senticosus* root extracts alone and their fixed combination, ADAPT-232**. **(A)** The number of unique genes deregulated by each extract alone and the number of deregulated genes that overlapped multiple extracts. **(B)** Pool of all genes whose expression was affected by any of the three extracts alone in comparison to ADAPT-232.

A microarray-based transcriptome-wide mRNA expression analysis was performed to identify possible targets of the tested substances in T98G cells. T98G cells were treated with test substances for 24 h in two technical replicates before total RNA was isolated and pooled for microarray hybridization. Significantly deregulated genes were identified compared to untreated controls (*p* < 0.05) by means of Chipster software analysis. Ingenuity Pathway analyses were performed with data sets of significantly up- or down-regulated genes: 536 genes for ADAPT-232 forte, 777 genes for ADAPT-232 forte tested at the low concentration, 534 genes for ADAPT-232 at the higher concentration, 591 genes for *Eleutherococcus* extract, 599 genes for *Schisandra* extract, 561 genes for substance *Rhodiola* extract, 567 genes for eleutheroside E, 620 genes for schizandrin B, 640 genes for salidroside, 601 genes for triandrin, and 562 genes for tyrosol. The Venn diagrams in Figure 2 shows the number of unique deregulated genes for each treatment extract in comparison to the number of common deregulated genes. Table 4 shows the main cellular functions and networks most significantly affected by various adaptogens. Tables 5 and 6 show genes up- or down-regulated by adaptogens.

**Table 4:** **Cellular functions associated with genes and genetic networks that showed significant differences in expression after treatment with the test samples**.

Functions	Test samples
Antigen presentation	Low concentration of ADAPT-322 forte
Cancer	Tyrosol
Carbohydrate metabolism	Eleutheroside E
Cell cycle	Salidroside
Cell death	ADAPT-232 forte, schizandrin B, tyrosol
Cell growth and proliferation	ADAPT-232
Cell–cell signaling and interaction	ADAPT-232 forte, *Schisandra* extract, schizandrin B, ADAPT-232, *Rhodiola* extract, eleutheroside E
Cellular assembly and organization	Low concentration of ADAPT-322 forte, schizandrin B
Cellular compromise	Schizandrin B
Cellular development	ADAPT-232, *Rhodiola* extract, *Schisandra* extract
Cellular movement	ADAPT-232, ADAPT-232 forte, salidroside
Inflammatory disease	Triandrin
Lipid metabolism	All tested substances except schizandrin B
Molecular transport	Triandrin, tyrosol
Nervous system development and function	Tyrosol
Neurological disease	Salidroside
Nucleic acid metabolism	*Eleutherococcus* extract
Small molecule biochemistry	*Eleutherococcus* extract, *Schisandra* extract, *Rhodiola* extract, eleutheroside E, triandrin, tyrosol
Vitamin and mineral metabolism	*Schisandra* extract

**Table 5:** **Genes up- and down-regulated by adaptogens in T98G cells**.

Genes	Genes family	Ligand	ADAPT-232 forte (300 mg/L)	ADAPT-232 forte (3 mg/L)	ADAPT-232	*Eleutherococcus* extract	*Schisandra* extract	*Rhodiola* extract	Eleutheroside E	Schizandrin B	Salidroside	Triandrin	Tyrosol
*GPR1*	Orphan							−3,294					
*GPR4*	SPC/LPC	lysoPL		51,268				3,482					
*GPR6*	orphan A1	sphingosine 1P	−3,434										
*GPR12*	orphan A1	sphingosine 1P		86,823	4,084			4,287	3,340				
*GPR17*	Orphan			125,366	−2,462	−2,445	−2,676			−2,990		−2,639	−2,676
*GPR18*	Orphan				−3,555		−6,589		−4,170		−3,555		
*GPR22*	Orphan											−2,412	
*GPR37*	orphan A4		−6,233		−3,411	−2,969		−5,464	−2,639	−3,411	−5,205	−4,141	
*GPR55*	Orphan		−2,514					−3,294					
*GPR52*	Orphan A2			190,019									
*GPR61*	Orphan			126,238									
*GPR65*	SPC/LPC				−6,869	−3,095							
*GPR78*	Orphan A3									−11,959			
*GPR82*	Orphan						−2,990						
*GPR83*	Orphan A9		−2,639				2,770				−7,210		
*GPR85*	Orphan SREB										−2,479		
*GPR88*	Orphan							−2,990		−2,770			−2,657
*GPR101*	Orphan					−2,428							
*GPR110*	LNB7TM									−8,754			
*GPR111*	LNB7TM		6,543			2,282	2,395	3,053					
*GPR114*	LNB7TM												−5,657
*GPR112*	LNB7TM				−3,630	−3,918	−2,928	−3,972	−2,282	−4,563			
*GPR128*	LNB7TM		3,681		3,555	2,908	5,242		3,891	2,908	3,053	3,458	
*GPR141*	Orphan		5,540				2,751			6,821	4,595	2,694	4,287
*GPR148*	Orphan		3,972		3,010	3,411	6,277	3,531		3,249		3,458	5,205
*GPR150*	Orphan										3,317		
*GPR151*	Orphan		−2,639		−6,869								
*GPR160*	Orphan							−3,434					
*GPR171*	Orphan							−3,434					
*GPR179*			2,313				−2,329						
*GPR182*			2,346										
*GPRC6A*	Calcium sensor		2,266		3,972	6,409	9,448		3,095		3,918	3,580	3,837
*GPR37L1*	Orpahn A6							2,888					
*HTR1F*	5-HTA	Serotonin								3.4			
*HTR1A*	5-HTA	Serotonin											−2.9
*GRM6*	Metabotropic glutamate	Glutamate	2.3		2.5	3.1	3.0		2.7		2.5	3.5	

*The values show fold changes compared to the control*.

**Table 6:** **Common genes up- and down-regulated by adaptogens and corresponding proteins in T98G cells**.

Gene and corresponding protein	*ADCY2* adenylate cyclase	*PDE4D* phospho-diesterase	*PI3KC2G* phosphatidyl inositol-3-kinase PIK3	*PRKCG* and *PRKCZ* protein kinase C	*PLCB1* phospholipase C beta 1 (phosphoinositide-specific)	*PLCD4* phospholipase C*	*CETP* cholesterol ether trans	*ESR1* estrogen receptor 1	*SERPIN* A**	*SERPIN* B**	*HTR3D* 5-HT_3_ receptor of serotonin	*HSPA1B* heat shock protein 72	*FOXA1*
ADAPT-232 forte (300 μg/mL)	−2.8	+4.3	+5.1		+4.7	−6.1	−8.9	−12.0	A3 −4.4		−4.3		
ADAPT-232 (172 μg/mL)	−2.9		+6.1		+3.7	−12.7	−7.6	−19.3	A1 + 9.3	B9 −3.3			
*Eleutherococcus* (30 μg/mL)	−2.4	+5.0	+2.7		+3.7	−13.2	−6.1	−22.6	A1 + 2.7	B2 −3.3 B4 −3.1	−2.2	3.0	+6.9
*Schisandra* (100 μg/mL)		+2.7	+7.2		+4.4	−9.6	−5.1	−5.8	A1 + 3.6	B2 −2.4 B3 −3.3	−3.4		
*Rhodiola* (40 μg/mL)	−3.7	+4.0	+6.6			−5.1	−9.8	−7.5	A1 + 12.7	B2 −4.5 B4 −3.4 B9 −4.2		+3.3	
Eleutheroside E (0.67 μM)			+3.2		+3.1	−10.1	−8.3	−7.1		B9 −2.7	−2.2		
Schisandrin B (5 μM)		+3.2			+4.1		−10.6	−3.4					P4 −21.7
Salidroside (3 μM)	−2.8	+4.2			+5.0	−10.1	−6.3	−3.0	A1 + 5.4	B2 −4.3	−6.6	+3.3	
Triandrin (1.5 μM)					+3.4	−5.1	−9.5	−2.9	A1 + 6.5	B2 −3.0 B4 −2.4		+3.6	RBFOX1 FOX1− 2.5
Tyrosol (3 μM)	−3.1	+4.6			+3.0	−9.9	−5.9	−6.6		B9 −2.6	−6.3		
ADAPT-232 forte (3 μg/mL)	*ADCY4*	+96.3		+67.2					A7 + 132.5			+44.0	+440
		+ 44.5		+115.4					A10 + 38.3			B8 +44.9	

*The values show fold changes compared to control*.

*(+) Indicates up-regulation*.

*(−) Indicates down-regulation*.

*PLCD4 expression is a marker for cancer. **Effects of PKC are cell-type specific: in CNS activation of PKC was associated with neuronal excitation (5-HT) and memory. **A1-A10 and B2-B9 represent the different isoforms of the SERPINs.

To exemplarily validate the microarray-based mRNA expressions, real-time RT-PCR reactions were performed for five genes using each two different pairs of treated and untreated samples. The expression of these genes were quantified and the ratios between treated and untreated samples were calculated and compared to the ratios obtained from microarray experiments (Table 3). The correlation coefficient between mRNA expression values determined by microarray hybridization and real-time RT-PCR was *R* = 0.81, indicating a high degree of concordance between results obtained by both methods.

## Discussion

Based on the analysis of results obtained in this study, we can draw several conclusions discussed in detail below.

### The effect of adaptogens on membrane-bound G-protein-coupled receptors

**Figure 3:**
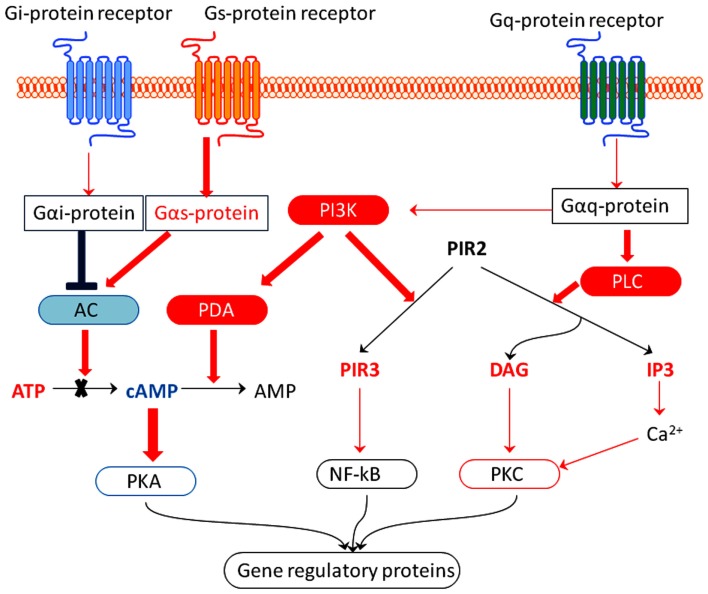
**Hypothetic molecular mechanisms by which adaptogens activate adaptive stress response pathways**. Neurons normally receive signals from multiple extracellular stressors that activate adaptive cellular signaling pathways, e.g., many neurotransmitters activate GTP-binding protein coupled receptors (GPCR). The receptors in turn activate kinase cascades including those that activate protein kinase C (PKC), activate protein kinase A (PKA), and phosphatidylinositol-3-kinase (PI3K). Effect of adaptogens on G-protein-coupled receptors pathways: up-regulated genes are represented in red, down-regulated in blue color. The G_s_ alpha subunit (or Gs protein) activates the cAMP-dependent pathway by activating adenylate cyclase. G_i_ alpha subunit (or G_i_/G_0_ or Gi protein) inhibits the production of cAMP from ATP. DAG, diacylglycerol; IP3, inositol triphosphate; PLC, phospholipase C.

A common feature of all drugs tested is their effect on G-protein-coupled receptor (GPCR) signaling pathways. All tested adaptogens significantly deregulated the expression of a large number of genes encoding (a) GPCRs (Table 5) and (b) key proteins of G-protein downstream pathways, i.e., cAMP, phospholipase C (PLC), and phosphatidylinositol mediated signal transduction (Table 6; Figure 3).

G-protein-coupled receptors (Gilman, 1987; Foord et al., 2005) are involved in a wide variety of physiological processes (Wettschureck and Offermanns, 2005; Hazell et al., 2011) including:

- Regulation of behavior and mood including binding of neurotransmitters such as serotonin, dopamine, GABA, and glutamate.
- Sympathetic and parasympathetic nervous systems, which are regulated by GPCR pathways, and are responsible for control of many automatic body functions such as regulating blood pressure, heart rate, and digestive processes.
- Regulation of immune system activity and inflammation
- Neuroendocrine homeostasis modulation.

All of these physiological processes are associated with stress and adaptogen activity, which increases stress tolerance.

The regulation of GPCRs is thought to play a fundamental role in maintaining physiologic homeostasis during stress (Carman and Benovic, 1998). A number of pathologic states are associated with disturbances in the functional activity of certain GPCRs (Lefkowitz, 1996). GPCRs are extremely important targets for neuropsycho-pharmacology (Roth et al., 1998). Many neuropsychiatric drugs either bind directly to specific GPCRs (e.g., antipsychotics) or affect GPCRs indirectly by influencing the amount of available native agonist (e.g., antidepressants; Von Zastrow, 2002).

Table 6 shows that adaptogens down-regulated the *HTR1A* gene encoding the 5-HT_3_ GPCR, which is known to activate an intracellular second messenger cascade resulting in excitatory or inhibitory neurotransmission (Hoyer et al., 1994). Activation of serotonin receptors modulate the release of many neurotransmitters including glutamate, GABA, dopamine, epinephrine/norepinephrine, and acetylcholine, as well as many hormones including oxytocin, prolactin, vasopressin, cortisol, corticotropin, substance P, and others. Activation of serotonin receptors also influence various biological and neurological processes such as aggression, anxiety, appetite, cognition, learning, memory, mood, nausea, sleep, and thermoregulation. A variety of pharmaceutical agents and illicit drugs, including many antidepressants and antipsychotics, target serotonin receptors (Yakel, 2000; Kennett et al., 1997). The binding of serotonin to the 5-HT_3_ receptor in neuronal cells of the central (e.g., brain-stem cells) and peripheral nervous systems opens the membrane channels, which in turn leads to an excitatory response and anxiety (Kennett et al., 1997). Thus, the observed down-regulation of the *HTR1A* gene by tyrosol (Table 6) is in line with publications that have demonstrated the anti-depressive effects of tyrosol in rats (Wikman and Panossian, 2004; Panossian et al., 2008) and recent publications showing that *Rhodiola* extracts affect serotonin receptors (Chen et al., 2009b; Mannucci et al., 2012). Serotonin receptors are also known to regulate longevity (Murakami and Murakami, 2008) and behavioral aging (Murakami et al., 2008) in the nematode *Caenorhabditis elegans*. This agrees with several publications demonstrating the beneficial effects of *R. rosea* on the lifespan of *C. elegance* and the fruit fly, *Drosophila melanogaster* (Jafari et al., 2007; Wiegant et al., 2009).

G-protein-coupled lysophospholipid receptors regulate cellular Ca^2+^ homoeostasis, cytoskeletal architecture, proliferation and survival, migration, and adhesion. They have been implicated in development; regulation of the cardiovascular; immune and nervous systems; inflammation; arteriosclerosis; and cancer. Lysophospholipid receptor ligands bind to and activate their corresponding transmembrane receptors. The activated receptors have a wide range of cellular effects depending on which ligand, receptor, and cell-type is involved. Primary effects include inhibition of adenylyl cyclase and release of calcium from the endoplasmic reticulum, while secondary effects include preventing apoptosis and increasing cell proliferation (Meyer zu Heringdorf and Jakobs, 2007). Down-regulation of GPCRs by adaptogens (Table 6) indicates that the number of receptors is greatly reduced leading to strongly attenuated signal transduction via G-proteins and their effectors (Von Zastrow, 2002), which may contribute to the beneficial effect of adaptogens in stress-related disorders.

G-protein-coupled receptor expression and G-protein-mediated signal transduction are affected by age and play an important role in the development of the major human pathologies associated with aging, cancer, neurodegenerative disorders, and cardiovascular diseases (Joseph et al., 1993; Alemany et al., 2007). This supports the results of *in vivo* studies in rats showing that ADAPT-232 has potential value for the treatment of age-related disorders of the stress system and may be effective in maintaining the health in elderly individuals (Makarov et al., 2007). ADAPT-232 counters effects of aging associated with:

- deregulation of programmed cell death (apoptosis),
- suppression of the immune system and spontaneous tumors promotion,
- decrease in liver detoxification,
- CNS malfunction (loss of memory and learning ability), and the development and progression of cardiac insufficiency, and hypercholesterolemia.

### The effect of adaptogens on cAMP-mediated G-protein signaling pathways

Taking into account that adaptogens down-regulated the expression of genes encoding adenylate cyclase and up-regulated gene expression for phosphodiesterase, we suggest that adaptogens reduce the cAMP level in brain cells by (a) inhibiting adenylate cyclase (inhibiting synthesis of cAMP) and (b) stimulating phosphodiesterase (stimulating degradation of cAMP; Figure 3).

Protein kinase A (PKA) acts in a cAMP-dependent manner (Walsh and Van Patten, 1994). Low levels of cAMP down-regulate PKA activity. PKA has several cellular functions including regulation of glycogen, sugar, and lipid metabolism. The effects of PKA activation vary with the cell-type, e.g., in adipocytes PKA stimulate lipases while in myocytes (skeletal muscle) and hepatocytes PKA increases glucose formation (by stimulating glycogenolysis and inhibiting glycogenesis) and its catabolic transformation to pyruvate (glycolysis). This provides free energy in the form of ATP and NADH, which play an important role in stress response.

The regulation of cAMP levels and PKA activity is a key mechanism of energy homeostasis and represents a metabolic switch between catabolism and anabolism (Walsh and Van Patten, 1994; Conti, 2000; Abramovitch et al., 2004; Vandamme et al., 2012). Down-regulation of cAMP and PKA by adaptogens decreases stress-induced catabolic transformations. PKA is presumably responsible for the stress-induced protective energy-saving effects of adaptogens. This energy-saving effect favors ATP-consuming anabolic transformations. Indeed, the inhibition of adenylate cyclase by adaptogens can increase intracellular ATP levels and prevent ATP from being converted to cAMP (Taussig and Gilman, 1995). This increased store of ATP might represent an energy source for other ATP-dependent enzymatic reactions required for metabolism.

Mitochondrial decay is a major cause of aging, leading to the subsequent death of aerobic organisms including humans. Impairments in mitochondrial antioxidant status were invariably associated with *decreased mitochondrial ATP-generation* capacities as well as with increases in mitochondria-driven reactive oxygen species (ROS) production in all tested tissues.

Long-term supplementation of male and female C57BL/6J mice (starting from the age of 36 weeks) with schizandrin B (administered at 0.012%, w/w) enhanced mitochondrial ATP-generation capacity in various tissues (brain, heart, liver, and kidney) during aging and mitigated age-dependent impairments in mitochondrial antioxidant capacity and functional ability, thereby retarding the aging process in mice, particularly during early aging, up to the age of 120 weeks (Ko et al., 2008). Schizandrin B was shown to increase the intracellular ATP levels and to protect against Gentamicin and Cyclosporine A-induced nephrotoxicity by decreasing oxidative stress and cell death *in vitro* and *in vivo* (Chiu et al., 2008; Zhu et al., 2012). *R. rosea* extract activated the synthesis or resynthesis of ATP in skeletal muscles mitochondria and stimulated reparative energy processes after intense exercise in rats. Treatment with *R. rosea* extract significantly (by 24.6%) prolonged the duration of exhaustive swimming in comparison with control rats (Abidov et al., 2003). Salidroside was shown to significantly increase the rate of ATP in isolated keratinocyte (Delepee et al., 2004). These studies are in line with our hypothesis on the mechanism of ATP-generation by adaptogens and their potential benefits in fatigue and aging-related diseases.

The brain’s prefrontal cortex cells are known to contain hyperpolarization-activated channels that can open when they are exposed to cAMP in stress. Excessive opening of these channels impairs cognitive function. It has been suggested that cAMP inhibitors can inactivate the channels allowing for normal neuron function. This reconnects the hyperpolarized cells to the neural network, and thus improves working memory, which plays a key role in abstract thinking, planning, organizing, etc. (Wang et al., 2007; Arnsten, 2011). Adaptogens may help in therapies for cognitive deficits associated with normal aging, and for cognitive changes associated with schizophrenia, bipolar disorder, or attention deficit hyperactivity disorder (ADHD; Wang et al., 2011).

This hypothesis is in accordance with our findings and publications, where ADAPT-232 and other adaptogens have demonstrated beneficial effects on cognitive function in humans (Bogatova et al., 1997; Hartz et al., 2004; Narimanian et al., 2005; Darbinyan et al., 2007; Fintelmann and Gruenwald, 2007; Weng et al., 2007; Olsson et al., 2009; Aslanyan et al., 2010; Panossian and Wikman, 2010; Edwards et al., 2012). Two clinical pilot studies showed that ADAPT-232 significantly improved attention and increased speed and accuracy during stressful cognitive tasks in comparison to a placebo control (Aslanyan et al., 2010). ADAPT-232 significantly decreased the number of mistakes made by test subjects in complicated psychometric tests (Bogatova et al., 1997) and improved the quality-of-life and recovery period of patients suffering from acute non-specific pneumonia (Narimanian et al., 2005).

### Adaptogen effects on G-protein signaling phosphatidylinositol and phospholipase C pathways

All tested adaptogens up-regulate the *PLCB1* gene, which encodes phosphoinositide-specific PLC and the *PI3KC2G* gene, which encodes phosphatidylinositol 3-kinases (PI3Ks; Table 6; Figure 3).

When activated by a G-protein, PLC catalyses hydrolysis of phosphatidylinositol 4,5-bisphosphate (PIP2) into diacylglycerol (DAG) and inositol-1,4,5-triphosphate (IP_3_; Figure 3; Gilman, 1987). IP_3_ is involved in a variety of cellular signaling pathways associated with phenomena as diverse as depression and tumor growth (Stutzmann, 2005; Sarkisov and Wang, 2008). DAG activates protein kinase C (PKC), which phosphorylates numerous other proteins (Nishizuka, 1995) and plays an important role in tumor growth (Yamasaki et al., 2009).

PI3K is a key upstream mediator of signal transduction and plays an important role in the regulation of NF-kB-mediated defense responses as well as apoptosis. PI3K is required for long-term enhancement of signal transmission between neurons, which is associated with potentiating memory and learning (Opazo et al., 2003; Karpova et al., 2006; Yang et al., 2008).

### Common effects on gene expression involved in regulation of cytoplasmatic and nuclear proteins

ADAPT-232 up-regulated the *SERPINI1* gene (serpin peptidase inhibitor, neuroserpin), which is involved in the development and function of the nervous system (Teesalu et al., 2004; Yepes and Lawrence, 2004). Neuroserpin controls axon growth and thereby supports the transmission of nerve impulses. It plays an important role in synapse development and regulates synaptic plasticity, suggesting that it may be important for learning and memory (Hastings et al., 1997; Davis et al., 1999; Kinghorn et al., 2006). Moreover, neuroserpin inhibits the activity of tissue plasminogen activator (tPA), which plays a role in cell migration, blood clotting, and inflammation (Yepes and Lawrence, 2004).

A characteristic feature common to all tested adaptogens was down-regulation of the *CETP* gene, which encodes cholesteryl ester transfer protein, a lipid plasma protein that facilitates the transport of cholesterol esters and triglycerides between low-density lipoproteins (LDL) and high-density lipoproteins (HDL; Bruce et al., 1998). Pharmacological inhibition of CETP alleviated atherosclerosis and other cardiovascular diseases, as well as metabolic syndrome (Barter et al., 2003).

Adaptogens down-regulated the *ESR1* gene (Table 6). *ESR1* encodes estrogen receptor alpha (ERα), a nuclear receptor belonging to a large family of transcription factors, transducing hormonal signals, and regulating expression of target genes (Evans, 1988). ERα is primarily localized in the cytoplasm (Welshons et al., 1984) in complexes with heat-shock-proteins (hsp90, hsp70, and hsp56) in a transcriptionally inactive form (Pratt, 1992). ERα are expressed in various tissues such as the breast, ovaries, testis, lung, uterus, bone, liver, and brain. In human forebrain, ERα are predominantly localized in amygdale (suggested to be involved in mood and cognition), hypothalamus (involved in learning and memory), and septum (Österlund and Hurd, 2001; Yaghmaie et al., 2005; Dahlman-Wright et al., 2006). Affective mood disorders, such as premenstrual syndrome, postnatal depression, and postmenopausal depression are associated with low serum-levels of estrogens. Estradiol treatment has been shown to down-regulate ERα in the uterus and in several brain areas (Shupnik et al., 1989; Lauber et al., 1990, 1991; Simerly and Young, 1991; Österlund et al., 1998).

ESR1 expression is tissue-specific and differentially regulated in a region specific manner in the brain. It has been shown that estrogen signaling via ERα can significantly attenuate an inflammatory neurodegenerative process by acting through astrocytes. ERα expression is necessary in astrocytes, but not neurons for neuroprotection in experimental autoimmune encephalomyelitis, the most widely used mouse model of multiple sclerosis – an autoimmune disease characterized by demyelination and axonal degeneration (Spencea et al., 2011). Consequently, it can be expected that neuroprotective effect of adaptogens (Kim et al., 2004; Bu et al., 2005; Chen et al., 2009a; Qu et al., 2009; Bocharov et al., 2010; Zhang et al., 2007, 2010; Li et al., 2011; Lee et al., 2012a,b; Palumbo et al., 2012; Shi et al., 2012; Zeng et al., 2012) must be associated with up-regulation ESR1 in glia cells. Surprisingly, the results of our study are not in line with this anticipation because adaptogens down-regulate ERα gene expression. How to interpret these contradictory observations – neuroprotection accompanied by down-regulation of ERα gene expression? This case is similar to another, when it was expected that stress-protective adaptogens have to reduce cortisol, because normally cortisol is increasing in stress. Indeed, adaptogens reduce cortisol in stress (Panossian et al., 1999b). However, adaptogens can increase secretion of cortisol/corticosterone when added to isolated adrenocortical cells (Panossian et al., 1999b; Panossian et al., 2007). Cell response (secretion of cortisol/corticosterone) to adaptogens and stress is similar – an activation, mild to adaptogens and much stronger in stress. However, stress response (secretion of cortisol/corticosterone) was significantly low in adaptogen pre-treated cells than in control cells (Panossian et al., 1999b; Panossian et al., 2007). Pretreatment with adaptogen, so-called “adaptive stress response” or “preconditioning” or “hormesis” (Mattson et al., 2007), acting as a mild stress or “stress vaccine,” adapts the cell to stress (Panossian et al., 1999b; Wiegant et al., 2008; Boon-Niermeijer et al., 2012). Similarly, it can be speculated that down-regulation of ERα gene expression by adaptogens is a signal for glia cell to initiate a feedback regulation of ERα. In a broader sense this concept is related to the inflammation, which is a defense response (“switch on” defense system) to cope the infection. In order to prevent an overreaction, a feedback mechanism of regulation of inflammation is activated, e.g., cortisol and anti-inflammatory cytokines are increasing (“switch off” defense system). Mild stress, in general, is a defense response in order activate innate immunity. In this context, adaptogens are natural substances initiating activation of the innate defense systems, including ERα as one of the component of stress system.

In addition to “classical” model of steroid receptor function, when the ligand-bound estrogen receptor regulates transcription of target genes in the nucleus by binding to estrogen response element regulatory sequences in target genes, estrogen activated membrane-bound CRα initiates rapid signaling PI3K/PLC pathways in astrocytes to indirectly modulate neuronal function and survival (Deroo and Korach, 2006; Mhyre et al., 2006). Estrogen treatment attenuates “non-classical” transcription at estrogen response elements sites in glioma cells (Mhyre et al., 2006). Adaptogens up-regulate *PLCB1*, *PI3KC2G*, and cAMP relate genes, modulate NO and SAPK. These similarities of estrogens and adaptogens allow to suggest that their mechanisms of actions someway interfere. Are they competing as mimetic or antagonists is still unclear albeit both are neuroprotective. Further study on elucidation of this mechanism of action of adaptogens is required.

ERs are over-expressed in around 70% of breast cancers (Deroo and Korach, 2006). Pharmacological down-regulation of *ESR1* may be effective in treatment and prevention of breast cancer, ovarian cancer, colon cancer, prostate cancer, and endometrial cancer. (Fabian and Kimler, 2005; Ascenzi et al., 2006).

Moreover, some of tested samples down-regulated the *OLFM4* gene, which was recently reported to inhibit apoptotic pathways and to promote tumor cell proliferation. This suggests that OLFM4 could serve as a diagnostic marker for human cancers (Koshida et al., 2007; Oh et al., 2011). Reduced expression of the *OLFM4* gene inhibits cell growth and increases sensitization to hydrogen peroxide and tumor necrosis factor alpha-induced apoptosis in gastric cancer cells (Liu et al., 2012). Down-regulation of the *PLCD4* gene (Table 6), which encodes cytoplasmatic PLC, is apparently associated with catabolic activity of adaptogens.

### The synergy/antagonism concept and considerations on dose-effect dependency

The mechanism of action of adaptogens in T98G cells was associated with deregulation of numerous genes. Even one single compound purified from the extracts, e.g., salidroside, triandrin, tyrosol from *Rhodiola*, schizandrin B from *Schisandra*, or eleutheroside E from *Eleutherococcus* affected the activity of 500–700 genes belonging to several different functional networks.

The approximate number of genes affected by any single compound in ADAPT-232 was on the same order as the number of genes affected by the entire mixture. Even if the gene expression profile contained the same genes (Figure 2), their expression remains of the same extent (the same fold range, Tables 5 and 6 and Supplementary Material). The total number of interactions in the molecular networks did not increase proportionally with the number of active compounds in the mixture. Further, the number of target genes affected by the complex mixture was decreased in some pathways as compared to single compounds. Figure 2 shows that 735 deregulated genes overlap between the single-component compounds. However, they do not account for all 1056 genes affected by ADAPT-232, suggesting that deregulation of 321 genes by ADAPT-232 is due to synergic interactions. Thus, it is possible that some of ADAPT-232’s pharmacological effects are associated with these 321 genes, supporting the synergy concept for herbal mixtures. On the other hand, the complete pool of all genes deregulated by three single extracts is 2188, while ADAPT-232 deregulates only 1056 genes. This indicates that antagonistic interactions of the three components in the mixture may result in suppression of some effects (possibly negative) of the mono-drugs in fixed combination in ADAPT-232. This is also in line with synergy/antagonism concept for herbal mixtures.

Purified single substances revealed differential regulation of more genes than did the extracts, e.g., *Rhodiola* extract affected more genes than salidroside, tyrosol, or triandrin, and ADAPT-232 affected more genes than *Rhodiola*, *Schisandra*, or *Eleutherococcus* extracts (Tables 5 and 6).

For example, schizandrin B at a concentration of 3 μM significantly down-regulated a number of genes (up to 12854 fold, Table 7) which were not affected in the total extract (in which the concentration of schizandrin was comparable). Moreover, the gene expression profiles of schizandrin B and *Schisandra* extract were quite different (Table 7). The same is true for other compounds as shown in Tables 6–8. This data may support the reductionistic concept of drug discovery common in “western medicine” that is based on the isolation and application of active constituents rather than the use of complex plant extracts.

**Table 7:** **Top down-regulated genes in schisandrin B-treated T98G cells as investigated by Ingenuity Pathway analysis**.

Symbol	Description	Fold change
**DOWN-REGULATED GENES**		
*XRCC6*	Ku70 protein*	−12854.6
*RPS21*	Ribosomal protein S21	−1618.0
*YPEL5*	Yippee-like 5 (Drosophila)	−1082.4
*KCTD20*	Potassium channel tetramerization domain containing 20	−252.5
*DSTN*	Destrin (actin depolymerizing factor)	−126.2

*Ku70 is a protein that, in humans, is encoded by the *XRCC6* gene, which is involved in DNA repair and chromatin remodeling. It is possible that *XRCC6* is involved in aging, although further results are necessary to determine whether it plays this role in humans. “Entrez Gene: XRCC6 X-ray repair complementing defective repair in Chinese hamster cells 6 (Ku autoantigen, 70 kDa).”

**Table 8:** **Top up-regulated genes in T98G cells treated with ADAPT-232 forte (3 mg/L) as investigated by Ingenuity Pathway analysis**.

Symbol	Description	Fold change
**UP-REGULATED GENES**		
*GCM2*	Glial cells missing homolog 2 (Drosophila)	+948.8
*EPHB1*	EPH receptor B1	+903.9
*ALX1*	ALX homeobox 1	+867.1
*CHRNB4*	Cholinergic receptor, nicotinic, beta 4 (neuronal)	+670.9
*SF3B2*	Splicing factor 3b, subunit 2, 145 kDa	+652.6
*WNT2B*	Wingless-type MMTV integration site family, member 2B	+584.1
*AICDA*	Activation-induced cytidine deaminase	+552.6
*CD37*	CD37 molecule	+505.0
*MDFI*	MyoD family inhibitor	+487.8
*TNNT2*	Troponin T type 2 (cardiac)	+461.4
*HSPA12B*	Heat shock 70 kDa protein 12B	+44.0
*HSPB8*	Heat shock 22 kDa protein 8	+46.8
*HTR5A*	5-Hydroxytryptamine (serotonin) receptor 5A, G-protein-coupled	+75.0

The dose at which the compounds were administered strongly influenced both the intensity of the cellular response and the profile of differentially expressed genes. For instance, ADAPT-232 forte taken at two different concentrations (3 and 300 mg/L) resulted in quite different gene expression profiles, including some instances of dose-dependent reversal effects on the same gene, e.g., the adenylate cyclase-encoding gene (Table 8).

In can be concluded that cellular sensitivity/reactivity is decreasing at higher doses of adaptogens resulted in increased cellular tolerance to other stressors. Some effects of individual ingredients on gene expression were neutralized when the ingredients were combined in mixtures. In general, only the most common effects attributed to the individual ingredients were observed in the complex mixtures. Interestingly, rather than potentiating gene expression levels, the combination of these components resulted in somewhat of a “smoothing effect” on gene expression level.

The limitations of any in virto cell models experiment obviously should be considered when conclusions are generalized. The changes in gene profile of *any isolated* cell line can not reflect all of those in *entire organism*, because of numerous interactions with circulating hormones and mediators of immune system. However, these *in vitro* experiments can be helpful in finding of possible key mechanisms of action of adaptogens, particularly when they are considered in the context of huge body of evidences obtained in clinical and preclinical studies. It is just the first step in order to define a realistic hypothesis. Further studies can be focused on evidences supporting this hypothesis. It is not excluded that plant extracts are metabolized in the liver thus, their metabolites may also compete with precursors. However, relevance of this *in vitro* model to the results of clinical studies is supported by pharmacokinetic studies of salidroside, tyrosol, eleutheroside E, and schizandrin. The half-life (*t*_1/2_) was 4.662 h for eleutheroside E (Feng et al., 2006), 4.5 h for salidroside in SHR-5 extract (Panossian et al., 2010), 2 h for tyrosol (Panossian et al., 2009), and 4.6 h for schizandrin (Wang et al., 2008). Pharmacokinetic curve of schizandrin has three maximums of concentration s in blood at 1, 3, and 8 h and it is still detectable in blood at least for 20 h (Wang et al., 2008; Liang et al., 2010). That is enough to reach brain from the bloodstream, to pass the blood-brain barrier and to interact with membranes of glial cells, where G-protein receptors are located.

In conclusion, the effects of adaptogens on gene expression profiles in neuroglial cells mainly affected energy metabolism (anabolic effects) and CNS functions. These results can be reconciled with beneficial effects of adaptogens in behavioral, mental, and aging-related disorders.

We conclude also, that blending two or more active substances in one mixture significantly changes deregulated genes profiles: synergetic interactions result in activation of genes that none of the individual substances affected, while antagonistic interactions result in suppression some genes activated by individual substances. Consequently, these changes can have an influence on transcriptional control of metabolic regulation both on the cellular level and the level of the whole organism. As a result, the pharmacological profile of the combination may differ from the profiles of the ingredients. Similarly, pharmacological profiles of purified compounds might differ from the pharmacological profile the total plant extract. Presumably, this phenomenon could be used to eliminate undesirable effects (e.g., toxic effects) and increase the selectivity of pharmacological intervention.

## Conflict of Interest Statement

Rebecca Hamm, Thomas Efferth, and Alexander Panossian declare no competing financial interests. Georg Wikman is a stockholder in the Swedish Herbal Institute (SHI).

## Supplementary Material

The Supplementary Material for this article can be found online at http://www.frontiersin.org/Neuroendocrine_Science/10.3389/fnins.2013.00016/abstract

## Footnotes

^1^http://www.chem.agilent.com/Library/usermanuals/Public/G414090040_GeneExpression_One-color_v6.5.pdf

^2^http://chipster.csc.fi/

^3^http://www.ingenuity.com/

^4^http://www.roche-applied-science.com/sis/rtpcr/upl/index.jsp?id = UP030000

^5^https://www.genscript.com/ssl-bin/app/primer

^6^http://www.ncbi.nlm.nih.gov/tools/primer-blast

## Abbreviations

FOXO, forkhead box O; HPA, hypothalamic-pituitary-adrenal; JNK1, c-Jun N-terminal protein kinase; NO, nitric oxide; NPY, neuropeptide Y.
